# Leaf phenology of thirteen African origins of baobab (*Adansonia digitata* (L.)) as influenced by daylength and water availability

**DOI:** 10.1002/ece3.4600

**Published:** 2018-10-26

**Authors:** Luisa Maddalena Di Lucchio, Rasmus Fensholt, Bo Markussen, Anders Ræbild

**Affiliations:** ^1^ Department of Geosciences and Natural Resource Management University of Copenhagen Copenhagen Denmark; ^2^ Laboratory for Applied Statistics, Department of Mathematical Sciences University of Copenhagen Copenhagen Denmark

**Keywords:** climate, daylength, dry‐zones, leaf flushing, origin, water stress

## Abstract

Water availability is often described as one of the main drivers of phenology of tropical dry forests but experimental studies to identify the cues triggering phenological changes are few. In a greenhouse trial, we exposed seedlings of thirteen origins, seven from West and six from East Africa, respectively, of *Adansonia digitata* L.(baobab) to a well‐watered control treatment and a water withholding treatment in combination with exposure to three different daylengths (11.5, 12.0, and 12.5 hr). Responses were measured in terms of meristematic activity, number of leaves, and height growth followed over 6.5 months. Multi‐level mixed‐effects statistical models were used to analyze how environmental and inter‐population variables affected phenological behavior. Flushing was influenced by the daylength with the lowest degree of meristematic activity observed in the short daylength treatment. Daylength also influenced the number of leaves through an interaction with the water regime. The water regime influenced all variables through interactions with the origins. Seedlings subjected to water stress had higher meristematic activity, but initially lower numbers of leaves than continuously watered plants. Height growth in continuously watered plants was fastest or at par with water‐stressed plants, depending on the origin. Origins from West Africa tended to have higher meristematic activity and their phenology was found to be less influenced by water withholding than East African origins. There were no signs of significant differences between origins in their response to photoperiod. In conclusion, baobab seedlings show opportunistic behavior, setting leaves when water is available, but larger activity is found when days are long. We discuss the results in terms of triggering factors for baobab phenology and adaptation to specific environmental conditions at the site of origins.

## 1 INTRODUCTION

Phenology (derived from Greek words phaino meaning to bring light and logos meaning study) is generally described as the analysis of the timing of life cycle phases or activities in plants and animals in relation to their expected potential drivers (Lieth, 1974). For trees, the annual timing of leaf flush and flowering is usually controlled by environmental cues, such as spectral composition of the light, length of the photoperiod, temperature, and water availability (Beck, Heim, & Hansen, 2004; Oquist & Huner, 2003).

In temperate and boreal ecosystems, many phenological phases are a function of temperature (Badeck et al., 2004), explaining why plants respond to the rising temperature and changing climate in general. For example, in many forests of the world (Wolkovich et al., 2012), the earlier leafing dates of trees and shrubs are interpreted as a response of the organisms to the phenomenon of the rising temperature (Polgar & Primack, 2011; Polgar, Gallinat, & Primack, 2014). The time from leaf appearance to leaf senescence defines the length of photosynthetic activity. Therefore, an early flushing and a late leaf fall extends the length of photosynthetic activity, affecting water, carbon and nutrient cycling (Panchen et al., 2015), increasing the plant productivity, the carbon dioxide uptake, and the annual net ecosystem production (Richardson et al., 2010; Wu, Gough, Chen, & Gonsamo, 2013).

The seasonality of tropical tree phenology, especially in dry areas, appears to be mainly adapted to the periodicity, duration, and intensity of drought (Justiniano & Fredericksen, 2000; Singh & Kushwaha, 2006; Wallace & Painter, 2002). The impact of water availability is not yet fully understood, partly because there are several tree types that are adapted to seasonal drought in different ways (Borchert & Rivera, 2001). While deciduous species shed their leaves during the early dry season and flush around the onset of the rainy season (Borchert, 1994), evergreen species are characterized by a more irregular leaf turnover. Stem succulent trees shed their leaves during the early dry season without being water stressed (Borchert & Rivera, 2001). For most species, the photosynthetic activity is confined to the rainy season suggesting that it is mainly controlled by the water availability (Borchert, 1994). However, water availability appears not to be the main environmental trigger of leaf flushing in woody species in the central Sahel as in fact most Sahelian species produce new leaves before the beginning of the first rains (Seghieri, Carreau, et al., 2012; Seghieri, Do, Devineau, & Fournier, 2012). In southern Africa, a similar greening before the onset of the rainy season was observed by satellite (Ryan, Williams, Grace, Woollen, & Lehmann, 2016; Tian et al., 2018). This suggests that leafing in the dry tropical trees may be triggered by other cues. For example, the study on tropical stem succulent trees conducted by Borchert and Rivera (2001) indicated that the synchronous bud break and dormancy could be induced by a change in the length of photoperiod. Still, it is not clear how phenology in seasonally dry tropical forests is influenced by the environmental conditions (Giraldo & Holbrook, 2011). It can be hypothesized that for species with widespread distribution, trees from different origins are likely to have adapted to the specific environmental conditions of the local region, resulting in different reactions to environmental stimuli, and in different timing of their phenological events. Such differences have been observed previously for temperate tree species (Aitken, Yeaman, Holliday, Wang, & Curtis‐McLane, 2008; Benito Garzón, Alía, Robson, & Zavala, 2011). Common garden studies of African tree species indicate that performance in terms of growth vary between origins and in some cases show clinal variation with parameters as for example rainfall (Bayala, Ouédraogo, & Ong, 2008; Larwanou & Reij, 2011). To the best knowledge of the authors, no papers have addressed genetic variation in phenology of different origins of tree species in dry tropical Africa although there are examples where different populations have been observed in situ for example Vihotogbé, Berg, Bongers, Sinsin, and Sosef (2014). Korbo et al. (2012) studied the seasonal development in productivity of leaves for West and East African origins of baobab in a common garden test in Mali, arguing for evolution of a geographical race or ecotype of Western African populations of baobab. Information on cues in phenology may be extracted from long‐term records, but long time series of phenological data for Africa are particularly rare and almost absent for the region of West Africa (Seghieri, Do, et al., 2012). That such time series are rare is hardly surprising given the challenge of spatial scales and the lack of historical records necessary for assessing changes over time (Herrmann, Sall, & Sy, 2014). Similarly, although the development of satellite‐based detection of vegetation phenology has increased the availability of both long‐term satellite (Zhang, Friedl, Tan, Goldberg, & Yu, 2012) and ground‐based NDVI (Normalized Difference Vegetation Index) measurements (Hmimina et al., 2013) also in the dry tropical Africa, this approach has not yet been applied in the region at the appropriate spatial scale required to identify and single out the phenology of individual woody species. Besides, this method necessarily confounds genetic and environmental effects, leaving the extent of phenological adaptation between different populations unresolved. An alternative approach that we apply in this paper is to subject seedlings of the same woody species but from 13 origins to different experimental treatments to reveal the nature of the triggering factors.

**Figure 1:**
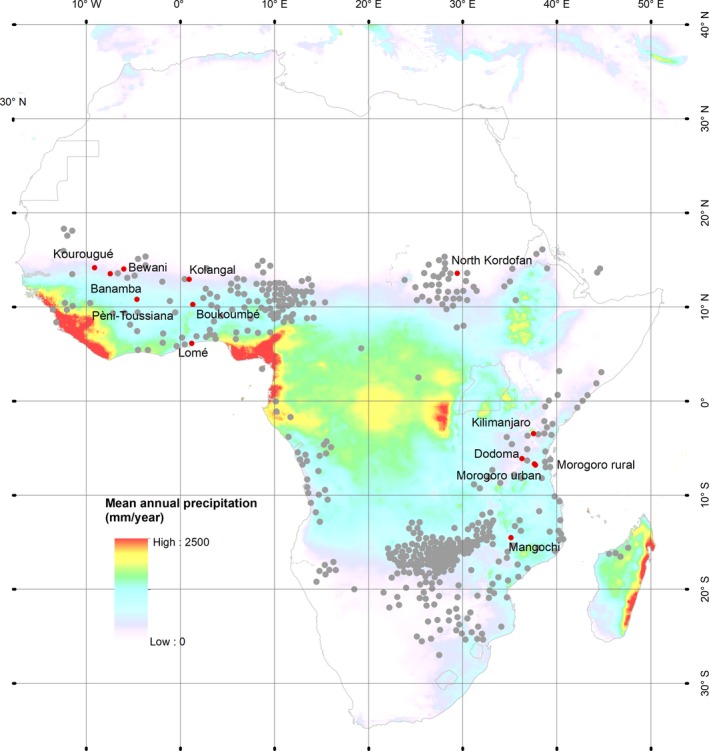
Distribution of Adansonia digitata in Africa (gray dots) according to Wickens (1982). The origins selected for the experiment are marked with red dots, and the background colors indicate the mean annual precipitation, based on the annual average of CHIRPS (Climate Hazards Group InfraRed Precipitation with Station data) precipitation 2000–2015

This paper investigates how leaf phenology of baobab is affected by the combined effects of drought and day length, using Africa's iconic baobab tree (*Adansonia digitata* L.) as a case study. Baobabs are drought resistant species and several studies have examined water use during the dry season for both adult trees (Chapotin, Razanameharizaka, & Holbrook, 2006, 2006; Fenner, 1980) and seedlings (Bouda, Bayala, Jensen, Markussen, & Ræbild, 2014; Van den Bilcke, De Smedt, Simbo, & Samson, 2013). In this study, we include plants from a large part of the distribution area from West to East of Sub‐Saharan Africa (Figure 1) to analyze whether the populations show signs of variation in the leafing phenology, and whether such variation can be related to the climate at the site of origin.

## 2 MATERIALS AND METHODS

### 2.1 Study species

*Adansonia digitata* L. (baobab) is a stem succulent and one of the best known tree species in semi‐arid Africa. The species is found in the Sudano‐Zambezian region with 200–800 mm annual rainfall, but also in areas with higher rainfall (up to 1,400 mm) (Wickens, 1982; Figure 1). The species is distributed from the West to the East Coast of the sub‐Saharan Africa, generally not occurring on deep sandy soils. In the north, it borders the semi‐desert scrub and grassland communities, whereas in the south, it occurs as far as the valleys of the Zambesi and Limpopo basins in South Africa (Figure 1).

Under most natural conditions, baobabs have leaves and produce flowers during the wet season and are leafless during most of the dry season (Wickens, 1982). The dry season coincides with the short days both north and south of the equator. Before the onset of rain, stem water supports the first flush of leaves (Chapotin, Razanameharizaka, & Holbrook, 2006).

The leaves of the baobab are simple to digitate. Trees begin each season by producing simple leaves that are soon (after 1 week) followed by 2‐3‐foliolate leaves, ending up with leaves that are 5‐7(‐9)‐foliolate (Wickens, 1982).

### 2.2 Selection of the origins

The seeds of *Adansonia digitata* L. selected for this trial were collected at 13 sites (origins) in 7 African countries (Benin, Burkina Faso, Malawi, Mali, Sudan, Tanzania, and Togo (Figure 1)) by project partners around 2005 and subsequently stored at a temperature of 4°C. The seeds were collected from 15 mother trees at each origin and kept separately for each mother tree. The origins span a range of environmental conditions characterized by annual precipitation from 650 to 1,050 mm, and maximum daylengths at the origins varied from 12.2 to 12.9 hr (Table 1).

**Table 1:** Origins of *A. digitata* used in the experiment. Annual precipitation, length of dry period, mean annual temperatureand the altitude elaborated represent estimates from New loc‐clim‐1.10, while daylight information was obtained from the UNL (University of Nebrasca‐Lincoln) Astronomy‐Daylight Hours Explorer

Location	Country	Latitude	Longitude	Altitude (m)	Annual rainfall (mm)	Dry days	Mean annual temperature (°C)	Maximum daylength (hr)	Minimum daylength (hr)
*West*									
Kourougue	Mali	14.17	−9.12	260	670	269	27.8	12.8	11.2
Banamba	Mali	13.55	−7.45	370	712	244	26.7	12.8	11.2
Bewani	Mali	14.04	−6.01	282	611	265	27.3	12.8	11.2
Peni‐Toussiana	Burkina Faso	10.82	−4.61	460	1,034	215	26.6	12.6	11.4
Kolangal	Burkina Faso	12.95	0.93	240	596	261	28.9	12.8	11.2
Lome	Togo	6.137	1.21	60	864	184	26.6	12.4	11.6
Boukoumbe	Benin	10.27	1.32	480	1,238	167	26.8	12.6	11.4
*East*									
North Kordofan	Sudan	13.58	29.42	560	390	304	26.7	12.8	11.2
Mangochi	Malawi	−14.51	35.14	180	784	247	26.0	12.9	11.1
Dodoma	Tanzania	−6.11	36.29	1,021	547	235	22.2	12.4	11.6
Kilimanjaro	Tanzania	−3.45	37.53	745	783	287	23.3	12.2	11.8
Morogoro rural	Tanzania	−6.78	37.73	480	868	164	24.9	12.4	11.6
Morogoro urban	Tanzania	−6.68	37.61	540	708	183	24.42	12.4	11.6

### 2.3 Seed pre‐treatment and germination

A germination test prior to the establishment of the trial showed that seeds had a germination rate down to 40%. Therefore, 30 seeds were selected per mother tree (in order to obtain ~12 seedlings) and placed in 0.4 L plastic cups, soaked in boiled water and left to cool overnight (Sacande, Rønne, & Sanon, 2006).

In June 2013, the seeds were sown in a greenhouse at 3 mm depth in trays (60 × 35 × 10 cm) that could host around 200 seeds (among 6 and 7 mother trees). The soil consisted of 10% sand mixed with 90% peat soil with added NPK and micronutrients, wetted with warm water. The seedlings attained an average height of almost 10 cm after 1 month and were transplanted to 10 cm diameter plastic pots with drainage holes. The same mixture of soil and sand was used for the transplanting.

### 2.4 Trial design

The study was carried out in a tropical greenhouse at Frederiksberg Campus, University of Copenhagen. Plants were arranged on six tables in two rooms (three tables in each). Starting from February 2014 (see below), the three tables in each room were subjected to three different daylengths. On each table, two water regimes were applied (see below), and within each water regime on all tables, origins were represented by 10 individuals from 10 mother trees, distributed randomly. Thus, each of the six tables contained 13 origins ×10 plants ×2 water regimes. The total number of plants was 1,560 for the whole experiment.

### 2.5 Environmental conditions and treatments

The seedlings were sprayed once with Movento Spirotetramat‐ Bayer CropScience Limited (5 ml/10 L) in September 2013, at the beginning of the experiment, to prevent attacks from aphids. Afterward, biological control was applied every two‐three weeks with predators of aphids, thrips, and mites.

Dataloggers (Onset HOBO Ware Pro, Bourne, MA) were used to measure temperature and monitor daylength, while the relative humidity was logged by the Priva climate control system (Priva E‐Measuring Box, Ontario, Canada). The measures were taken at 5 min intervals throughout the period of experiment.

The air temperature was set to a minimum of 18°C and went up to 34°C max during the day and 29°C max during the night. The relative humidity varied between 19% and 100% in both rooms.

Until October 2013, all plants were watered once per week. Starting from October 25 (day of year [doy] 298), two water regimes were applied. The plots of control plants were irrigated once per week (called “constant”), while the plots of the water‐stressed plants were irrigated every three weeks (called “water stressed”). The water stress treatment led to loss of turgor in leaves and stems of the trees, loss of leaves and the apparent collapse of the plants. In order to prevent widespread mortality, weekly watering of the water‐stressed plants was resumed from mid‐January 2014 (doy 16) and continued until the experiment ended in June, 2014.

In parallel until October 2013, the plants were growing under the natural light regime. From 23 October (doy 298) to 25 February 2014 (doy 56), the seedlings were grown under a 10‐hr photoperiod using supplementary lamps (Philips MASTER SON‐T PIA Plus 600W/220 E40 1SL) switched on from 7:00 a.m. until 5:00 p.m. The assumption was that, if phenology was controlled by photoperiod, the plants would enter into a state of meristemal rest. After February 25, when natural daylengths exceeded 10 hr, three daylengths were applied to the three tables in each room, letting the plants grow under short (11.30 hours), medium (12.00 hours), and long (12.30 hours) photoperiods. In order to obtain the short and medium daylengths, these tables were protected with automated black curtains to block light pollution from the lamps of the rooms. As there were no curtains used in the long photoperiod treatment, daylength was continuously increasing after March 26th where the natural daylength exceeded 12.5 hr.

### 2.6 Data collection

Measurements were taken in the months of December, January‐February, March, May and June and dates expressed as day of year. The height, presence/absence of developing leaves or buds (hereafter called meristematic activity) and number of leaves were assessed for each plant at six occasions. Total height was measured from the ground level to the top of the developing bud. For resource reasons, only approximately 125 plants per table were assessed in December, and the height variable was not measured during the last assessment in June. The meristematic activity was registered in the last four assessments. A plant was coded “1” for meristematic activity if any of its buds showed signs of growth and “0” if no buds were growing. Counting of the number of leaves took into account the leaf differentiation described by (Wickens, 1982), and leaves were counted only when they were sufficiently developed to allow a visual identification of their morphology. From the first assessment, the plants differentiated into simple and 2‐3‐4‐5‐foliolate leaves. The number of each type of leaf was counted on each plant at all assessments except the last, where only the total leaf number was counted.

### 2.7 Statistical analysis

Statistical analysis was performed using R with the nlme, MASS, lsmeans, and lme4 (Lenth, 2015; Pinheiro, DebRoy, Sarkar, & Core Team, 2015; Venables & Ripley, 2002) packages. The meristematic activity, the number of leaves (calculated as the sum of each group of *n*‐foliolate leaves), and height were modeled separately, using the individual plant data set.

For meristematic activity, a logistic regression with random effects model was applied to the binary variable (presence/absence of meristematic activity) in the lme4 (Bates, Mächler, Bolker, & Walker, 2015) package. The model included all interactions of the four effects of water availability (2 levels), daylengths (3 levels), origins (13 levels), and day of year (4 levels). Since fitting of random effects of table and room was not possible, only a random effect of plant was included. The resulting model for the *i* ^th^ individual had the form:

(1) $$\text{logit}\left(\text{Prob}\left(\text{activity for individua}l_{\left(i\right)}\right)\right)=\alpha_{\text{Wr}\left(i\right),\text{daylength}\left(i\right),\text{Origin}\left(i\right),\text{doy}\left(i\right)}+\;B\left(\text{plan}t_{i}\right)$$

where logit (Prob(activity for individual_(_ *_i_*_)_)) is the probability of presence/absence of meristematic activity, coded as 1 or 0. Fixed effects α_Wr(_ *_i_*_),daylength(_ *_i_*_),Origin(_ *_i_*_),doy(_ *_i_*_)_ represent the set of regression coefficients for each treatment and their interactions as stated above. *B*(plant_i_)represent the random variation between plants. To generate *p*‐values for each model coefficient, we used repeated measures of ANOVA. Because budburst was not assessed in the first two assessments we redefined the data set to exclude these assessments from the analysis.

For number of leaves and height growth, we used a linear mixed‐effects model (Dai et al., 2004) with nested random effects (Laird & Ware, 1982), applying interactions among the four explanatory variables used to model the meristematic activity, as described above, including the effect of room (2 levels).

A Gaussian spatial residual correlation with a nugget effect (Diggle model) was included where the day of the year denotes the range. The final model was:

(2) $$\text{Log}\left(1+\text{Leave}s_{i}\right)=\alpha_{\text{Wr}\left(i\right),\text{daylength}\left(i\right),\text{Origin}\left(i\right),\text{doy}\left(i\right),\text{room}\left(i\right)}+B\left(\text{bloc}k_{i}\right)\;+\;C\left(\text{plan}t_{i}\right)+\varepsilon_{\text{month}\left(i\right),\text{plant}\left(i\right)}$$

where Log(1 + Leaves*_i_*) is the response for plant *i* (either the number of leaves or the height growth), and the fixed effects α_Wr(_ *_i_*_),daylength(_ *_i_*_),Origin(_ *_i_*_),doy(_ *_i_*_),room(_ *_i_*_)_ represent the set of regression coefficients for each treatment and their interactions. The random effects B(block*_i_*) and C(plant*_i_*) allow the regression intercepts to vary, and the error terms ε_month(i),plant(i)_ are correlated across months within plants. The response variables were transformed to achieve linearity and homoscedasticity, using Box‐Cox transformations. A constant (+1) was added to the response variable before the transformation was applied, as Box‐Cox Power transformations only work if all data are >0 (Box & Cox, 1964). Because the height was not measured at every assessment, the corresponding missing values were removed from the analysis. Visual inspection of residual plots did not reveal any obvious deviations from homoscedasticity or normality. *p*‐values were obtained by Chi‐square tests of the effects in the chosen model, and adjusted means for each effect were estimated using the lsmeans package (Lenth, 2015).

Finally, we used simple graphical analyses to investigate links between origin, climate, and performance.

## 3 RESULTS

### 3.1 Meristematic activity

**Figure 2:**
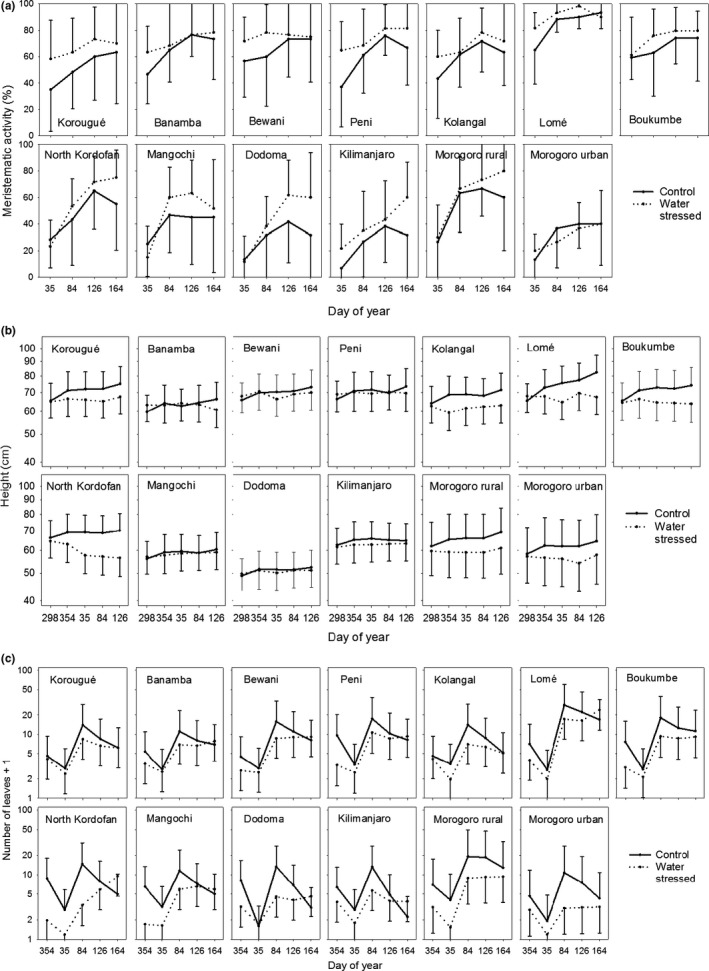
Least square means and 95% confidence intervals for the interactions between water regime, origin, and day for three observed response variables: A percentage of plants showing meristematic activity, B height growth, and C number of leaves. The lines show the model responses to water availability (dotted line: water stressed; solid line: control); Within each panel, the upper row represents origins from West Africa, while the lower row represents East Africa. Within each row, origins are organized from West to East

A general increase in the fraction of plants having meristematic activity was observed from doy 35 to the end of the experiment, with the most noticeable increase between day 84 and day 126 and in some origins a decrease from day 126 to day 164 (Figure 2a).

Random effects explained a considerable part of the variation, of which the largest part was due to variation between plants (Table 2). Hence, even within the origins of the same treatments, a large variability in the response was observed.

**Table 2:** Results from statistical analyses for the three variables

1. Estimated variance components of random effects		
Random effects	Estimated variance	Proportion of total variation
*Meristematic activity*		
Plant	2.9099	1.0
*Height growth*		
Block	0.0000	0.00
Plant	0.0466	0.80
Residual	0.0192	0.20
*Number of leaves*		
Block	0.0561	0.05
Plant	0.1260	0.11
Residual	0.9934	0.85

2. Significance tests of significant systematic effects			
Effect	LR‐statistic	DoF	*p*‐value
*Meristematic activity*			
Day of year: Daylengths	140.62	6	<0.0001
Water regime: Day of year: Origin	129.18	72	<0.0001
*Height growth*			
Water regime: Daylengths: Room	5.44	2	0.0659
Water regime: Day of year:Origin	101.85	48	<0.0001
Water regime: Day of year: Room	16.08	4	0.0029
Daylengths: Day of year: Room	23.20	8	0.0031
*Number of leaves*			
Location: Room	26.71	12	0.0085
Water regime: Day of year:Daylengths	35.66	10	0.0001
Water regime: Daylengths: Room	5.20	2	0.0741
Water regime: Day of year:Origin	128.67	60	<0.0001
Water regime: Day of year: Room	36.31	5	<0.0001
Daylengths: Day of year: Room	297.41	10	<0.0001

There was a significant interaction between water regime, origin, and day of year, suggesting that the origins were affected differently by the water regime over time (Table 2). Exposure to water stress leads to a slightly larger probability of meristematic activity across all origins (*χ* ^2^ (1) = 23.63, *p* < 0.0001, Figure 2a). Origins (distributed as in Figure 1) from East Africa tended to have lower meristematic activity than those originating from West Africa. For plants that had been exposed to water stress, the origins from East and West Africa also followed a different temporal development, as plants from West Africa tended to have a higher level of meristematic activity at the onset of recording day 35 (Figure 2a). The origin from Togo (Lomé), which represents a relatively moist origin and is the only origin in the trial with two rainy seasons, had the highest level of meristematic activity both for plants exposed to water stress and for plants kept under constant water supply.

**Figure 3:**
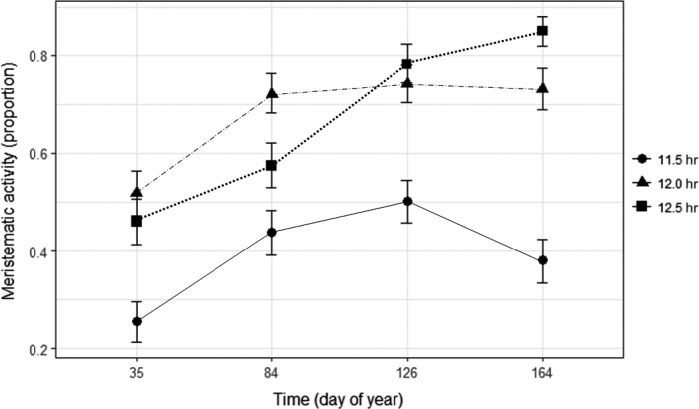
Development of meristematic activity over time in the three daylength treatments (means with error bars representing 95% confidence intervals). Closed circle: short daylength (11.5 hr). Closed triangle: medium daylength (12.0 hr). Closed square: long daylength (12.5 hr). Lines show the model responses to different daylengths (dotted line: 12.5 hr; dashed line: 12.0 hr; solid line: 11.5 hr

The significant interaction between daylength and day of year suggests that the three levels of daylength influenced the meristematic activity of the plants differently (Table 2). In the long day treatment (12.5 hr), the probability of meristematic activity constantly increased with day of year. In the medium daylength (12.0 hr) treatment, meristematic activity initially increased to stay at a relatively high level, while in the short‐day treatment, meristematic activity always stayed at a low level, although it increased until day 126 after which it decreased (Figure 3). The interaction between origins and daylengths was not significant (*χ* ^2^ (72) = 74.36, *p* = 0.41), suggesting that the triggering effect of daylength may be similar on all origins.

### 3.2 Height growth

The interaction between water regime, origin, and day of year was highly significant for height, indicating that growth of the plants in relationship to the two water treatments was different among the origins (Table 2). Under the steady watering in the control plants, height showed an increasing trend for most origins (Figure 2b). For the plants subjected to water stress, height growth was more modest, absent, or even negative due to death of parts of the stem.

The fastest height growth was found in the Lomè origin, and in general, the western origins were growing faster than the eastern origins under constant water supply (Figure 2b). We found no other evidence of geographical trends in growth.

Daylengths had only a limited effect through the interaction between water regime, daylength, and room (not shown), and the lack of interaction between daylength and origin suggested no difference between origins in the effect of daylength on growth (*p* = 0.748).

### 3.3 Number of leaves

**Figure 4:**
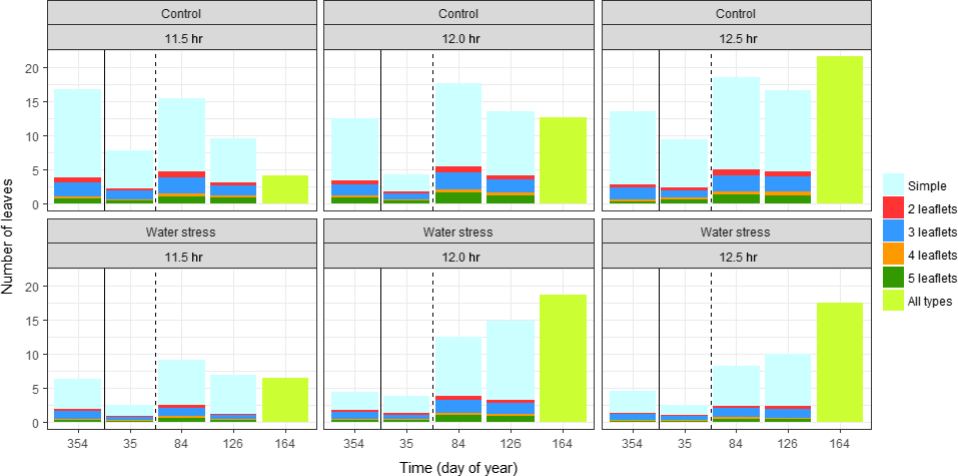
Effects of water regime and daylengths on number of leaves. Single and 2‐3‐4‐5 foliolate leaves (leaflets) were counted from doy 354 to doy 126, while at the last assessment only the total leaf number was recorded. The end of the water stress treatment is indicated by the solid line, while the initiation of daylength treatments is indicated by the dashed line

The baobab seedlings always had more single leaves than compound leaves, although the proportions varied (Figure 4).

Simple leaves also showed the largest changes. Across all treatments, the number of compound leaves tended to be largest at the assessments at doy 85 and 126. Analyzing the total number of leaves per plant, the selected model showed a significant interaction between water regime, daylength, and day of year (Table 2).

During the 10 hr photoperiod that was applied before the treatments started, the number of leaves decreased (doy 354 to doy 35). An additional reduction in number of leaves was seen in plants subjected to water stress, having on average only 2–3 leaves at doy 35. Approximately, 1 month after onset of the light treatments (doy 84), all treatments had larger numbers of leaves compared to doy 35, with the largest values obtained for plants under constant water supply. At doy 126, responses diverged with the number of leaves decreasing in all treatments, except for water‐stressed plants at 12.0 and 12.5 hr where the number of leaves increased. At the end of the trial (doy 164), the number of leaves had stabilized or increased for plants at 12.0 and 12.5 hr daylength. For the plants at 11.5 hr daylength, the number of leaves decreased or stayed low.

Since compound leaves have a larger leaf area than simple leaves (roughly proportionate to the number of leaflets), we also calculated the average number of leaflets per plant. This analysis essentially showed the same pattern, as the number of leaflets increased for water‐stressed plants at 12.0 and 12.5 hr treatments from doy 84 to doy 126, but decreased in all other treatments (not shown).

Seedlings from different origins reacted differently to water regimes over the trial period as shown by the significant interaction between water regime, origin, and day of year. Figure 2c (lower panel) illustrates that origins from the East African group tended to have larger differences between the two water regimes than the group of origins from West Africa (upper panel). The highest mean values occurred in the Lomé (Togo) and the Morogoro rural (Tanzania) origins with ~20 leaves per plant at doy 84 for plants under constant water supply, whereas the origin from North Kordofan (Sudan) subjected to water stress had the lowest amount of leaves per plant, many plants being leafless at doy 35. Even though there was no obvious correlation with latitude or longitude in the responses, analysis of contrasts showed that the western origins had higher number of leaves than the eastern origins, which was particularly the case in the last three assessments.

## 4 DISCUSSION

### 4.1 Effects of water stress and daylengths

Baobab seedlings show a complex phenology and to some extent seem to apply an opportunistic strategy, where leaf phenology is influenced both by daylength and water availability. Plants with a continuous water supply tended to have larger numbers of leaves than water‐stressed plants, not only during the water stress treatment but also when watering was resumed for all plants. It is well‐known how some deep‐rooted and stem succulent tropical species use stored water to maintain their leaves under drought conditions (Borchert, Rivera, & Hagnauer, 2002), and baobab seedlings have developed mechanisms to minimize water use and growth to survive drought events (Van den Bilcke et al., 2013). Water stress led to a loss of leaves, resulting in a smaller transpirational area and thus a reduction of transpiration, although the loss of stem rigidity indicated that transpiration was continuing from the stem surface. When watering was resumed the seedlings quickly responded by a steep increase in the number of leaves (Figure 4). However, even in the drought treatment where there was occasional dieback of shoots, no population was ever completely without plants having leaves, and leaf shedding and flushing was not strongly synchronous. Although mature trees mostly show a distinct deciduous habit, there are examples of baobab trees in moist environments that keep leaves throughout the year (Wickens & Lowe, 2008). Likewise, Korbo et al. (2012) showed that baobab seedlings would produce leaves when watered regularly during the cold and dry season in a study in Mali. Yet in the same study, leaf production was low during the cold short‐day period and increased markedly during the warm and dry months of March, April, and May to culminate during the wet season. This indicates that baobab leaf phenology is affected by water availability in combination with temperature or daylength, but that the leaf area is regulated to be maximal during the rainy season.

The sensitivity of phenology to water availability vary between dry zone species. For example, (Elliott, Baker, & Borchert, 2006) showed that Dipterocarps in dry seasonal forests in Thailand were evergreen on low, moist sites and deciduous on high dry sites. On the other hand, (Bate & Franklin, 2015) showed that irrigation had no impact on leaf phenology in *Brachychiton megaphyllus*, a shrub from the northern Australian forests. With its phenology affected by both water availability and daylength, Baobab seems to belong to species that are more sensitive to changes in water availability. Unfortunately, our setup does not allow to determine whether water availability is a triggering factor for leaf phenology, or it is merely a resource controlling the magnitudes of responses to other cues.

Numerous coincidences between budbreak and the spring equinox have been documented for example (Elliott et al., 2006; Lawton & Akpan, 1968; Rivera et al.., 2002). Although such coincidences indicate that length of the photoperiod may be important, there is a risk that other, uninvestigated factors correlate with daylength, and therefore such claims need to be substantiated by experiments. Unfortunately, such experiments are rare for tropical tree species, although Borchert and Rivera (2001) subjected various tropical stem succulent trees to increased daylength and found that an increase in daylength of 30 min was sufficient to trigger phenological changes. In the current study, analysis of the meristematic activity confirms Borchert and Rivera (2001)’s finding that shoot growth seems to be favored by long daylengths. Moreover, daylength (through the interaction with water availability) had a significant impact on the number of leaves (Figure 4), even though the changes over time revealed a complex pattern. Finally, growing the plants at 10 h daylength at the initial stage of the experiment led to a dramatic reduction in the number of leaves. Although variations in daylengths close to the equator are much smaller (Table 1) than at higher latitudes, the seasonal differences thus appear to be sufficient to trigger phenological events including budbreak. Interestingly, Borchert et al. (2015) suggested that the cue for phenology could be an increase in insolation (defined as the total amount of solar radiation received) rather than in daylength, based on an analysis of a wide range of species of the American continents. We estimated insolation in the different daylength treatments (data not shown) by using data from an outdoor PAR (photosynthetically active radiation) sensor and measurements of the strength of the supplementary light. This revealed only modest differences between daylength treatments, as the accumulated insolation in the 11.5 hr treatment was approximately 94% of insolation in the 12.5 hr treatment (estimated from the start of the daylength treatments to the last records at doy 164). In addition, in the 11.5 hr treatment meristematic activity was decreasing at a time where the insolation was increasing strongly. This would suggest that differences among treatments are due to differences in daylength rather than to changes in insolation.

### 4.2 Differences between populations

Populations of baobab had different phenology in response to water availability as evidenced by the significant interactions between origin, water availability, and day of year. However, the observed difference between eastern and western populations in the meristematic activity immediately after release from stress (Figure 2a) represents the only clear geographical signal in the data that we could identify. A possible interpretation is that the East African populations have a deeper dormancy and are less responsive to water once stress is relieved. The difference between groups is supported by earlier observations of differences in growth and stress response between western and eastern populations (De Smedt et al., 2012; Korbo et al., 2012) and also by studies of chloroplast and ITS (Internal Transcribed Spacers) haplotypes, showing a major division between western and eastern/southern populations (Cron et al., 2016; Pock Tsy et al., 2009).

Within the sampled dry zone origins, rainy seasons north and south of the equator are essentially mirrored, corresponding to the summer in both hemispheres. For example, in the Sudanese location, the rainy season stretches from July to September, whereas in the tropical and sub‐tropical origins south of the equator, the rainy season is approximately from December to March. This may explain the large similarities in phenology between northern and southern origins. However, it is conspicuous that the origin from Lomé, which is the only population with a bi‐modal rainy season, had high meristematic activity, a large number of leaves and fast height growth. Due to the annual movements of the ITCZ (intertropical convergence zone), the rainy season in the coastal location of Lomé‐Togo is distributed from the end of April to July with a second and shorter rainy season in September and October, whereas for the inland sites only one rainy season from July to September is observed (WMO 2015). With its origin in a climate with less pronounced seasons, it could be a selective advantage to be more responsive to water availability. Similarly, Morogoro rural, the wettest origin in the East African group (Figure 1), had high levels of meristematic activity and number of leaves compared to other origins within the western group, confirming the opportunistic strategy of the species.

As the populations did not interact significantly with daylength, there is no evidence that they respond differently to the daylengths. This suggests that they are either triggered by a common signal (e.g. daylengths ≥12 hr), or that the changes in daylength triggering differences are smaller than the 30 min increments applied in our treatments. More detailed studies are required to resolve which of the two hypotheses are correct.

## 5 CONCLUSIONS AND OUTLOOK

Baobab phenology is affected by both water stress and daylength. Drought causes a loss of leaves, and when the water returns there is an increase in meristematic activity that continues if daylength is long (12 hr or more). The daylength hence triggers increased numbers of leaves but this effect was found to have less effect under humid conditions. The seedlings reacted differently depending on their origin, but only in their reaction to water availability. The question is whether or not the phenology of other, non‐succulent species from dry seasonal areas will be controlled by the same cues. Several functional types have been identified with respect to leaf phenology, and it could be interesting to examine deciduous species with various leafing periods, semi‐evergreen species, and evergreen species (Borchert & Rivera, 2001; Seghieri, Do, et al., 2012). Examples of such species from the study area include *Acacia Senegal* (L.) which has a deciduous leaf habit but occasionally flushes leaves before the onset of the rainy season, and *Parkia biglobosa* (Jacq.) G.Don which is almost evergreen, but sheds leaves, is flowering and has leaf flush in the middle of the dry season. This seems especially relevant in times of climatic change, where species whose phenology is controlled by daylength may make them less likely to respond to changes in water availability.

## AUTHOR CONTRIBUTIONS

LMD and AR conceived the ideas; LMD and AR conducted the greenhouse trial and collected the data; LMD and BM analyzed the data; LMD led the writing with assistance from AR and RF.

## DATA ACCESSIBILITY

The data associated with this publication are deposited at UCPH ERDA data repository and can be accessed at https://dx.doi.org/10.17894/ucph.3328c507-664b-4101-89e9-6257e943b8c0.
